# The influence of protective psychosocial factors on the incidence of dental pain

**DOI:** 10.11606/s1518-8787.2022056004061

**Published:** 2022-07-04

**Authors:** Mariana Guimarães Jorge de Alvarenga, Maria Augusta Bessa Rebelo, Gabriela de Almeida Lamarca, Janice Simpson de Paula, Mario Vianna Vettore

**Affiliations:** I Universidade Federal de Minas Gerais Departamento de Odontologia Social e Preventiva Belo Horizonte MG Brasil Universidade Federal de Minas Gerais . Departamento de Odontologia Social e Preventiva . Belo Horizonte , MG , Brasil; II Universidade Federal do Amazonas Faculdade de Odontologia Manaus AM Brasil Universidade Federal do Amazonas . Faculdade de Odontologia . Manaus , AM , Brasil; III University of Agder Department Health and Nursing Sciences Kristiansand Agder Norway University of Agder . Department Health and Nursing Sciences . Kristiansand , Agder , Norway

**Keywords:** Child, Toothache, epidemiology, Sense of Coherence, Psychosocial Support Systems, Health Education, Dental, Longitudinal Studies

## Abstract

**OBJECTIVE:**

To investigate the influence of protective psychosocial factors on the incidence of dental pain in the last six months among 12-year-old children living in Manaus (AM).

**METHODS:**

A prospective school-based cohort study was conducted with 210 12-year-old students enrolled in public schools in the eastern zone of Manaus (AM). Students were followed up for two years. Validated questionnaires were used to assess sociodemographic characteristics, protective psychosocial factors, including sense of coherence, social support, and self-esteem at baseline and after two years. Calibrated examiners clinically assessed dental caries and gingival bleeding. Multivariate multilevel Poisson regression was used to estimate the relative risk (RR) and 95% confidence interval (95%CI) between the changes on psychosocial factors scores and incidence of dental pain, adjusted for psychosocial factors scores at baseline, dental health insurance, frequency of tooth brushing, and dental caries.

**RESULTS:**

Mean scores for sense of coherence and social support reduced significantly from baseline to 2-year follow-up. The incidence of dental pain along the two-year follow-up was 28.6%. The risk of dental pain was 14% higher for every 10 points in the mean reduction of sense of coherence score (RR = 1.14; 95%CI: 1.02–1.20), and 6% higher for every 10 points of the mean reduction in social support score (RR = 1.06; 95%CI: 1.01–1.11). Change on self-esteem was not associated with risk of dental pain.

**CONCLUSION:**

Change on sense of coherence and social support over the two-year period influenced the incidence of dental pain among children, suggesting that protective psychosocial factors, health behaviours, dental health insurance, and clinical oral condition have an important role in the incidence of dental pain.

## INTRODUCTION

Pain is a subjective event that is related to physiological, psychological, social, and cultural factors. Dental pain derives from changes in the teeth or their supporting structures, and untreated dental caries is its main biological cause ^1^ . Several factors such as cariogenic diet, poor oral hygiene, low exposure to fluoride, and low socioeconomic status have been associated with dental caries ^2^ .

Psychological factors may exacerbate the perception of pain due to catastrophic thoughts of pain, anxiety and fear associated with pain. These are the main psychosocial factors investigated with regards to dental pain ^3^ . Protective psychosocial factors may be defined as the absence of or low exposure to a risk factor. Although protective psychosocial factors are conceptually distinct from risk factors, the former may affect health independently or mitigate the effects of risk factors on health ^4^ . Protective psychosocial factors such as sense of coherence (SOC), social support, and self-esteem may modulate the perception of pain, and favour coping with risk events ^5^ .

The salutogenic theory is based on problem-solving orientation and the ability to identify and effectively use the available resources to improve health. It proposes an expanded understanding of the determinants of health by highlighting the importance of population’s health promoting factors and quality of life. SOC is considered the core construct of salutogenesis, also comprising the general resources of resistance, including social support and self-esteem ^5^ . SOC is also defined as an orientation for people to perceive life as comprehensive, manageable, meaningful ^5 , 6^ . Social support is the provision of psychological and material resources intended to help individuals cope with stress, understand that they are cared for, loved, members of a collective ^7 , 8^ . Self-esteem bears interrelated aspects that involve the sense of personal efficacy and personal value, being the integrated sum of self-confidence and self-respect, and the belief that the individual is competent to live and worthy of living ^9^ .

Studies on the role of psychosocial protective factors on oral health, especially dental pain, are scarce. The current study aimed to investigate the influence of protective psychosocial factors, including SOC, social support, and self-esteem, and the incidence of dental pain over the last six months in 12-year-old children.

## METHODS

A prospective longitudinal study was conducted with schoolchildren, parents and guardians in the East Zone of the city of Manaus (AM). A stratified random sample of 12-year-old children, regularly enrolled in the 7th grade, was selected from 25 municipal public schools in 11 neighbourhoods in the East Zone of Manaus. The selection of the schools was proportional to the number of schools in each neighbourhood. Children with any syndrome and/or with special care needs, and those undergoing orthodontic treatment were excluded. This research was approved by the ethics and research committee of the *Universidade Federal do Amazonas* (CAAE 57273316.1.0000.5020).

A theoretical model with a hierarchical structure among its components was adapted to test the influence of protective psychosocial factors on dental pain. According to this model, sociodemographic characteristics would influence access to dental health insurance, frequency of dental visits, frequency of tooth brushing, and protective psychosocial factors, which would have an effect on oral clinical measures, including dental caries and gingival bleeding, which in turn would influence dental pain (  Figure  ) ^10^ .

**Figure:**
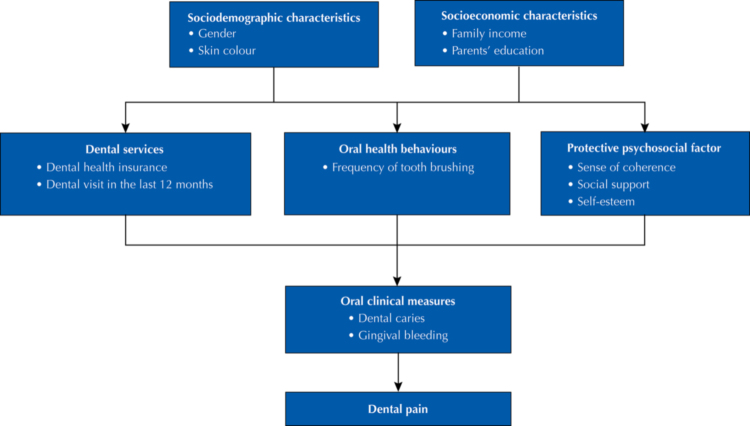
Theoretical model on the predictors of dental pain adapted from Bastos et al. 10 (2005).

Data collection at baseline was conducted from September to December 2016 with a self-completed questionnaire comprising closed-ended questions about demographic and socioeconomic characteristics, dental health insurance, and use of dental services, and was answered by the children’s parents/guardians. In addition, children completed a self-administered questionnaire to assess dental pain and tooth brushing frequency, and completed SOC, social support and self-esteem scales validated for the Brazilian population at baseline and after two years. Examiners recorded the decayed, missing and filled teeth index (DMF-T) and gingival bleeding through clinical examination using a plain dental mirror No. 5 (Duflex ^®^ ), ball point WHO probe, and personal protective equipment. Clinical oral examination was performed by five (baseline) and seven (two-year follow-up) previously calibrated examiners. Children were examined with privacy under natural light in classrooms chosen by the Board of each school. Kappa coefficients for inter-examiner calibration of the DMF-T index ranged from 0.914 to 0.988.

Dental pain in the last six months was assessed through the question: “In the last six months did you experience dental pain?” using the response answers “yes” or “no”. Pain intensity was assessed using the Faces Pain Scale - Revised (FPS-R) ^11^ , according to the version adapted to Portuguese ^12^ . The FPS-R is a self-rated pain faces scale, developed to measure the intensity level of perceived pain based on six faces presented horizontally and representing different degrees of pain, from “no pain” to “a lot of pain”.

Demographic and socioeconomic characteristics included gender (male/female), skin colour (White/Black/Yellow/Brown/Indigenous), monthly family income in minimum wages (MW) (≤ ½ MW/> ½ to 1 MW/> 1 MW), and parents’ education (assessed according to the completed years of education with approval). Frequency of tooth brushing (< 3 times a day/≥ 3 times a day), dental health insurance (yes/no), and dental visit in the last 12 months (yes/no) were assessed.

SOC was assessed with the SOC-13 scale ^5 , 13^ . The questionnaire comprises 13 questions that are answered on a 5-point Likert scale. The scores of the questions that are contrary to the SOC are inverted to obtain the final score that is obtained by summing the scores of the 13 items. The higher the score, the higher the SOC.

Social support was assessed using the Social Support Appraisals (SSA) instrument, developed specifically for children ^14^ . The questionnaire consists of 30 6-point Likert scale with the following response options: fully agree, strongly agree, agree a little, disagree somewhat, strongly disagree, and fully disagree. The final SSA score vary from 30 to 180, and the higher the score the greater the perceived social support.

Self-esteem was evaluated using the Self-esteem Scale, which ranks the level of self-esteem into low, medium, or high ^15 , 16^ . The original scale was developed for adolescents, and has ten closed-ended questions, five of which refer to positive “self-image” or “self-worth”, and five refer to “negative self-image” or “self-depreciation”. Items are answered on a four-point Likert scale ranging from strongly agree, agree, disagree, and strongly disagree. Higher scores on the Self-Esteem Scale indicate a higher self-esteem.

Dental caries was evaluated according to the DMF-T index, which measures the experience of dental caries on permanent dentition ^17^ . For this purpose, healthy teeth (code 0), filled teeth without caries (code 3), missing teeth (codes 4 and 5), and teeth with sealants (code 6) were recoded as “0”, and teeth evaluated as decayed (code 1) and restored with caries (code 2) were recoded as “1”. The modified codes were then summed to obtain the number of decayed teeth for each participant. The number of teeth with gingival bleeding was calculated according to the presence or absence of bleeding on probing. In each participant, a randomly selected upper quadrant and the corresponding contralateral lower quadrant were examined. The presence of gingival bleeding was recorded on all faces of all teeth in the selected quadrants based on bleeding on probing component of the Community Periodontal Index (CPI) ^18^ .

Firstly, the prevalence of dental pain at baseline, and the incidence of dental pain and the respective 95% confidence intervals (95%CI) were estimated. The sample was described based on measures of proportions and means with 95%CI for independent variables, for the sample at baseline and at the two-year follow-up, and according to the incidence of dental pain. Change in the frequency of tooth brushing was assessed according to the difference between the number of children who reported brushing their teeth “three times or more a day” from baseline to follow-up. The differences in the SOC-13, SSA, and Self-esteem Scale scores between baseline and follow-up were employed to estimate the change on SOC, social support, and self-esteem, respectively.

Correlations between SOC, social support, and self-esteem at baseline and two-year follow-up in children was assessed by the Spearman’s correlation coefficient. The frequency of tooth brushing at baseline and two-year follow-up was compared with McNemar’s test. Comparisons of SOC, social support, self-esteem scores, and the number of decayed and gingival bleeding were evaluated through the Wilcoxon test for paired samples.

Multivariate multilevel Poisson regression was used to estimate the relative risk (RR) and 95%CI between independent variables and incidence of dental pain. Multilevel analysis was employed because of the two-stage sampling process: schools and children. The association between the scores of the SOC, social support, self-esteem scales and the incidence of dental pain were estimated for the change in every 10 points of the scale ^19^ . Independent variables with p-value < 0.10 in the crude analysis were considered for the multivariate analysis. Because of the significant correlations among the psychosocial factors, separate multilevel Poisson models were employed to test the association between each psychosocial factor and the incidence of dental pain adjusted for covariates.

## RESULTS

The baseline sample consisted of 288 children. During the 2-year follow-up period of the study, 78 children were not reassessed due to loss to follow-up. Thus, the analysed sample included 210 schoolchildren assessed after two years of follow-up. Of them, 150 did not report dental pain in the last 6 months, and made up the control group. The group with incidence of dental pain was composed of 60 students.

The incidence of dental pain after the two-year follow-up was 28.6%; most of the schoolchildren with dental pain selected the faces one and two, which suggest low intensity of the perceived dental pain (76.7%), the intermediate pain (faces three and four) was chosen by nine children (15%), while the “a lot of pain” face was selected by five children (8.3%).

Among the children with incidence of dental pain, 60% were female, 73.3% were of brown skin colour, and 45% from families with monthly income ranging from half to one minimum wage. Most participants with incidence of dental pain after the two-year follow-up did not have dental health insurance (90%), had been to the dentist in the last year (60%), and brushed their teeth less than 3 times a day (51.7%). The mean scores of SOC, social support, and self-esteem of participants with incidence of dental pain were 47, 145.8, and 31.5, respectively. The mean scores of SOC, social support, and self-esteem showed no meaningful differences between the total sample and the studied sample at baseline ( Table 1 ).

**Table 1:** Demographic and socioeconomic characteristics, dental services, psychosocial factors, tooth brushing, and oral clinical measures at baseline, for the total sample and the study sample, and according to dental pain in the last six months at the two-year follow-up in children.

Variable	Baseline data from the study sample (n = 210)	Dental pain in the last 6 months at 2-year follow-up	
		Yes (n = 60)	No (n = 150)
	Mean (95%CI)	Mean	Mean (95%CI)
Dental pain at 2-year follow-up	28.6 (22.9–35.1)	100.0	0.0
**Demographic characteristics**			
Sex			
Male	45.2 (38.6–52.1)	40.0 (28.3–52.6)	47.3 (39.4–55.4)
Female	54.8 (47.9–61.4)	60.0 (47.0–71.7)	52.7 (44.6–60.6)
Skin color			
White	16.1 (11.8–21.9)	13.3 (6.7–24.7)	17.3 (12.0–24.3)
Black	9.1 (5.8–13.8)	5.0 (1.6–14.6)	10.7 (6.6–16.7)
Yellow	3.8 (1.9–7.5)	5.0 (1.6–14.6)	3.3 (1.4–7.8)
Brown	68.1 (61.4–74.1)	73.3 (60.6–83.1)	66.0 (58.0–73.2)
Indigenous	2.9 (1.3–6.3)	3.4 (0.8–12.6)	2.7 (0.9–7.0)
**Socioeconomic characteristics**			
Monthly family income			
≤ ½ MW	25.8 (20.3–32.3)	21.7 (12.9–34.0)	27.5 (20.9–35.3)
> ½ to 1 MW	41.6 (35.1–48.5)	45.0 (32.8–57.8)	40.3 (32.6–48.4)
> 1 MW	32.5 (26.5–39.2)	33.3 (22.5–46.3)	32.2 (25.1–40.2)
Parents’ education, mean	11.1 (9.9–12.3)	11.9 (8.9–14.8)	10.8 (9.5–12.0)
**Dental care**			
Dental health insurance			
Yes	11.4 (7.8–16.5)	21.6 (12.9–34.0)	7.3 (4.1–12.8)
No	88.6 (81.4–95.2)	90.0 (84.0–93.9)	75.0 (62.4–84.4)
Dental visit in the last 12 months	44.3 (37.7–51.1)	0.60 (0.47–71.7)	47.3 (39.4–55.4)
**Tooth brushing**			
Frequency of tooth brushing			
< 3 times a day	35.7 (29.5–42.5)	51.7 (39.0–64.1)	29.3 (22.6–37.2)
≥ 3 times a day	64.3 (57.5–70.5)	48.3 (35.9–61.0)	70.7 (62.8–77.5)
Protective psychosocial factors			
Sense of coherence, mean	45.8 (45.1–46.5)	47.0 (45.4–48.7)	46.1 (45.2–47.1)
Social support, mean	144.1 (141.8–146.5)	145.8 (141.3–150.3)	143.5 (140.8–146.2)
Self-esteem, mean	31.7 (30.9–31.9)	31.5 (30.5–32.4)	31.2 (30.8–31.9)
Clinical oral measurements			
Dental caries, mean	0.5 (0.4–0.7)	0.8 (0.5–1.1)	0.4 (0.2–0.6)
Gingival bleeding, mean	3.3 (3.0–3.7)	3.3 (2.5–4.1)	3.4 (2.9–3.8)

MW: minimum wages; 95%CI: confidence interval of 95%.

All correlations between the psychosocial variables at baseline and between psychosocial variables at the two-year follow-up were statistically significant. At baseline, the strongest correlation was between social support and self-esteem (coefficient = 0.419; p < 0.001), while for the two-year follow-up it was between SOC and social support (coefficient = 0.632; p < 0.001).

The frequency of tooth brushing ≥ 3 times a day reduced from 64.3% to 56.7% between baseline and two-year follow-up. Mean SOC and social support scores reduced significantly between both periods. The mean number of decayed teeth and teeth with gingival bleeding increased from 0.5 to 1.3, and from 3.3 to 8.2, respectively, between baseline and the two-year follow-up ( Table 2 ). These results suggest a worsening in the frequency of tooth brushing, SOC, and social support.

**Table 2:** Comparisons of psychosocial factors, frequency of tooth brushing, and clinical oral measures between baseline and two-year follow-up in children.

Variables	Baseline	2-year follow-up	p
	Mean (95%CI)	Mean (95%CI)	
**Dental brushing**			
Frequency of tooth brushing			0.002 ^a^
< 3 times a day	35.7 (29.5–42.5)	43.3 (36.7–50.2)	
≥ 3 times a day	64.3 (57.5–70.5)	56.7 (49.8–63.3)	
**Psychosocial protective factors**			
Sense of coherence, mean	45.8 (45.1–46.5)	42.9 (41.7–44.0)	< 0.001 ^b^
Social support, mean	144.1 (141.8–146.5)	138.5 (135.6–141.3)	0.001 ^b^
Self-esteem, mean	31.7 (30.9–31.9)	30.8 (30.2–31.3)	0.298 ^b^
**Clinical oral measurements**			
Dental caries, mean	0.5 (0.4–0.7)	1.3 (1.0–1.5)	< 0.001 ^b^
Gingival Bleeding, mean	3.3 (3.0–3.7)	8.2 (7.8–8.6)	< 0.001 ^b^

95%CI: confidence interval of 95%.

^a^ p refers to the McNemar’s test.

^b^ p refers to Wilcoxon’s test for paired samples.

The unadjusted relative risk measures and respective 95%CI between the independent variables and the incidence of dental pain in the last six months at the two-year follow-up are presented on Table 3 . The associations of dental pain incidence after the two-year follow-up with dental health insurance, change in the frequency tooth brushing, change on SOC, change on social support, and dental caries after the two-year follow-up had p-value lower than 0.10, and were included in the multivariate multilevel Poisson regression models. Self-esteem was not associated with the incidence of dental pain at the two-year follow-up. Thus, the variable was not tested in the multivariate multilevel Poisson regression model.

**Table 3:** Unadjusted associations between sociodemographic characteristics, dental services, tooth brushing, protective psychosocial factors, oral clinical measures, and dental pain incidence in children using multilevel Poisson regression.

Variable	RR	95%CI	p
**Demographic characteristics**			
Sex (ref.: Male)			
Female	1.24	0.74–2.10	0.416
Skin colour (ref.: White)			
Black	0.70	0.18–2.69	0.606
Yellow	1.69	0.44–6.54	0.449
Brown	1.34	0.63–2.88	0.449
Indigenous	1.49	0.31–7.18	0.621
**Socioeconomic characteristics**			
Monthly family income (ref.: > 1 MW			
> ½ to 1 MW	1.08	0.60–1.94	0.797
≤ ½ MW	0.83	0.41–1.69	0.609
Parents’ education	1.01	0.99–1.03	0.512
**Dental care**			
Dental health insurance (ref.: No)			
Yes	0.46	0.25–0.87	0.017 ^b^
Dental visit in the last 12 months (ref: No)			
Yes	0.96	0.57–1.60	0.864
**Tooth brushing**			
Frequency of tooth brushing (2 years) (ref.: < 3 times a day)			
≥ 3 times a day	0.88	0.53–1.47	0.616
Change on the frequency of tooth brushing	1.52	0.95–2.44	0.078 ^b^
**Protective psychosocial factors**			
Sense of coherence (baseline) ^a^	1.15	0.86–1.19	0.449
Sense of coherence (2 years) ^a^	0.87	0.69–1.03	0.111
Change in Sense of Coherence ^a^	1.13	1.01–1.19	0.035 ^b^
Social Support (baseline) ^a^	1.03	0.95–1.09	0.488
Social Support (2 years) ^a^	0.96	0.89–1.02	0.153
Change in Social Support ^a^	1.05	1.00–1.10	0.053 ^b^
Self-esteem (baseline) ^a^ .	1.04	0.63–1.21	0.825
Self-esteem (2 years) ^a^	0.84	0.51–1.12	0.312
Change in self-esteem ^a^	1.11	0.87–1.20	0.319
**Clinical oral measurements**			
Dental caries (baseline)	1.14	0.97–1.34	0.109
Dental caries (2 years)	1.11	0.99–1.24	0.075 ^b^
Change in dental caries	1.08	0.91–1.29	0.369
Gingival bleeding (baseline)	0.99	0.90–1.09	0.824
Gingival bleeding (2 years)	0.96	0.89–1.05	0.377
Change in gingival bleeding	1.02	0.95–1.09	0.579

RR: relative risk; 95%CI: 95% confidence interval; MW: minimum wages.

^a^ Estimates for each 10 points on the scale.

^b^ p < 0.10.

Multilevel multivariate Poisson regression models between protective psychosocial factors and the incidence of dental pain were analyzed separately for SOC and social support due to the observed collinearity between variables ( Table 4 ). Changing every 10 points on the SOC scale increased the risk of dental pain by 14% (RR = 1.14; 95%CI: 1.02–1.20). The incidence of dental pain was 6% higher for each 10-point increase in the change of the social support scale score (RR = 1.06; 95%CI: 1.01–1.11). Schoolchildren with dental health insurance had a lower incidence of dental pain than those without dental health insurance. In addition, the risk of dental pain in the last two years was higher among children who changed the frequency of tooth brushing compared to those who did not.

**Table 4:** Multilevel Poisson regression models between dental health insurance, change in frequency of tooth brushing, SOC and social support, dental caries, and incidence of dental pain.

Variable	RR	95%CI	p
**Sense of coherence (adjusted model)**			
Dental health insurance (ref.: No)			
Yes	0.49	0.26–0.91	0.024
Change on the frequency of tooth brushing	1.61	1.01–2.57	0.046
Sense of coherence at baseline ^a^	0.96	0.68–1.15	0.745
Change on sense of coherence ^a^	1.14	1.02–1.20	0.030
Dental caries at 2-year follow-up	1.10	0.99–1.22	0.087
**Social support (adjusted model)**			
Dental health insurance (ref.: No)			
Yes	0.47	0.25–0.87	0.017
Change on the frequency of tooth brushing	1.69	1.04–2.76	0.035
Social Support at baseline ^a^	0.99	0.90–1.06	0.780
Change in social support ^a^	1.06	1.01–1.11	0.038
Dental caries at 2-year follow-up	1.08	0.97–1.20	0.172

SOC: sense of coherence; RR: relative risk; 95%CI: 95% confidence interval.

^a^ Estimates for each 10 points on the scale.

Independent variable estimates are adjusted for each other.

## DISCUSSION

This study evaluated the possible effect of changes in protective psychosocial factors, including SOC, social support, and self-esteem, on the incidence of dental pain among children over a two-year period. Significant reductions were observed in SOC and social support between baseline and after two years of follow-up. The SOC reduction may be related to the social status of the participants, since a previous study reported that SOC was lower in adults with worse social indicators ^20^ . Self-esteem did not vary between the study periods. Changes on SOC and social support increased the risk for incidence of dental pain in children.

This study confirmed the importance of access to dental health insurance and of behaviours, including frequency of tooth brushing, for the occurrence of dental pain ^2^ . However, the association between dental caries and dental pain was not observed in the current study, although it has been reported in previous studies ^1 , 2^ . A possible explanation for this result was the use of the DMF-T index to assess caries. The DMF-T index does not assess the severity of caries, only its presence or absence. Thus, the outcome may be due to the fact that a significant proportion of decayed teeth in the sample was composed of early dental caries lesions.

Studies on the possible influence of psychosocial factors on dental pain in children are scarce. A previous systematic review demonstrated that fear and anxiety related to dental care are common in children and adolescents, and that these psychological factors were related to dental pain ^3^ . Moreover, stress and anxiety were predictors for expectation, perception and memory of dental pain in children ^21^ . Anxiety was associated with the onset of orofacial pain during a two-year follow-up ^22^ . Evidence for the influence of psychosocial factors on dental pain is scarce, considering the salutogenic theory ^23^ .

There is evidence on the relationship of protective psychosocial factors with oral health outcomes and use of dental services. Higher maternal SOC increased the likelihood of their children using dental services for preventive and check-up reasons ^24^ . A recent systematic review highlighted the relationship between protective psychosocial factors, including maternal SOC, self-efficacy, and social support, and lower likelihood of dental caries in children and adolescents ^4^ . The association between maternal SOC and lower caries occurrence in children has also been demonstrated ^25^ . Furthermore, SOC, self-esteem, and health beliefs in adolescents were direct predictors for untreated dental caries and oral health-related quality of life ^26^ . In another study, greater social support was associated with better clinical oral health status in adolescents ^27^ . Social support had an indirect effect on dental pain, and this relationship was mediated by clinical oral condition. Social support also influenced oral health-related quality of life, and perceived oral health ^27^ . Thus, the reported association between psychosocial factors and dental pain may be explained by the positive effects of protective psychosocial factors on the use of dental services, and oral clinical conditions.

Although self-esteem was not associated with dental pain in this study, self-esteem attenuated the impact of malocclusion on oral health-related quality of life in children with less orthodontic treatment needs. Moreover, self-esteem was associated with oral health-related quality of life ^9^ . One may suggest that self-esteem is a relevant psychosocial factor for subjective oral health measures, but might not enough to influence dental pain.

The possible relationship between psychosocial factors and dental pain has been evaluated in cross-sectional epidemiological studies. Longitudinal research involving protective psychosocial factors and oral health outcomes were reported regarding the relationship between SOC, social support and oral health beliefs, oral health-related quality of life, and oral health-related behaviours ^26 , 28^ . No longitudinal study was found on protective psychosocial factors and dental pain. Thus, the need for further longitudinal studies on this topic is highlighted to increase the understanding of the determinants of dental pain, including the mediating and moderating role of psychosocial factors, and on the relationship of socioeconomic and clinical characteristics with dental pain. A previous study showed that SOC, although not directly related to dental pain, played a relevant moderating role in the association between dental caries and dental pain ^23^ .

The search for problem solving, the use of resources that drive the individual towards healthier health-related behaviours, a sense of coherence that makes one perceive life as manageable and meaningful are forms of modulation performed by the SOC in the light of the salutogenic approach. Thus, a strong SOC enables people to maintain oral health care even in the face of stressful life situations ^6^ . Social support, through the establishment of social connections, provides emotional, material, and psychological resources to face adverse and stressful situations ^8^ . Mutual relationships between individuals may also positively influence health behaviours through control and social pressure, and give rise to a feeling of responsibility, which motivates and strengthens self-care and mutual care among members of social groups. For example, being part of a social network in which members have oral hygiene and healthy eating habits influences the subject to follow the same healthy behaviours of the group, thus having positive impacts on health ^7^ . These elements reinforce the importance of the salutogenic approach and its principles on the grounds for formulating health promotion policies and strategies.

Most collective actions in oral health propose only individual and collective preventive measures, such as the increase in the access to the use of fluoride through fluoride toothpaste and fluoridation of water supply to prevent dental caries. Moreover, individual interventions focused on behavioural changes prevail, including the increase in the frequency of tooth brushing and improvement of tooth brushing techniques. The implementation of oral health promotion strategies, combined with the salutogenic perspective, may be considered incipient. A recent systematic review showed that interventions on social networks positively modified behaviours, including smoking reduction, and perception of well-being ^29^ . An intervention study found that SOC increase resulted in the improvement oral health-related quality of life in children ^30^ . Thus, future intervention studies should address the possibility of reducing the perception of dental pain by changing protective psychosocial factors.

However, some limitations of this study should be considered. The study sample included children aged 12 years at baseline, who were followed-up for two years. So, the findings should not be considered for other age groups. Participants were selected in an area of social inequality. Thus, caution is recommended when extrapolating the results to populations living in different social conditions. Finally, some behavioural factors related to dental pain proposed in the theoretical model adopted in this study, including use of toothpaste and flossing, were not assessed ^9^ .

Among the protective psychosocial factors investigated in this study, SOC and social support scores reduced between baseline and the two-year follow-up. A reduction in tooth brushing frequency was also observed. Change on SOC and social support significantly increased the risk of dental pain along the two-year follow-up. Reduced frequency of tooth brushing and lack of dental health insurance also influenced the incidence of dental pain. The findings of this study suggest that protective psychosocial factors, health behaviours, and dental health insurance play an important role in the incidence of dental pain in children.
