# Supplementary material for: The influence of protective psychosocial factors on the incidence of dental pain
Source: Rev Saude Publica. 2022 Jul 4;56:67. doi: 10.11606/s1518-8787.2022056004061 (PMC9274973; doi:10.11606/s1518-8787.2022056004061)
Supplement: Translation

# Translation: A influência de fatores psicossociais protetores sobre a incidência de dor dentária

**Authors:** Mariana Guimarães Jorge de Alvarenga, Maria Augusta Bessa Rebelo, Gabriela de Almeida Lamarca, Janice Simpson de Paula, Mario Vianna Vettore

**Affiliations:** I Brasil Universidade Federal de Minas Gerais. Departamento de Odontologia Social e Preventiva. Belo Horizonte, MG, Brasil; II Brasil Universidade Federal do Amazonas. Faculdade de Odontologia. Manaus, AM, Brasil; III Norway University of Agder. Department Health and Nursing Sciences. Kristiansand, Agder, Norway

**Keywords:** Criança, Odontalgia, epidemiologia, Senso de Coerência, Sistemas de Apoio Psicossocial, Educação em Saúde Bucal, Estudos Longitudinais

## Abstract

**OBJETIVO:**

Investigar a influência de fatores psicossociais protetores sobre a incidência de dor dentária nos últimos seis meses em crianças de 12 anos residentes em Manaus (AM).

**MÉTODOS:**

Um estudo de coorte prospectivo de base escolar foi realizado com 210 alunos de 12 anos, matriculados em escolas públicas da zona leste de Manaus (AM) que foram acompanhados por dois anos. Questionários validados foram usados para avaliar características sociodemográficas, fatores psicossociais protetores, incluindo senso de coerência, apoio social e autoestima na linha de base e após dois anos. Examinadores calibrados avaliaram clinicamente cárie dentária e sangramento gengival. Regressão de Poisson multinível multivariada foi usada para estimar o risco relativo (RR) e o intervalo de confiança de 95% (IC95%) entre a variação dos escores dos fatores psicossociais e a incidência de dor dentária, ajustada para os escores dos fatores psicossociais na linha de base, plano de saúde odontológico, frequência de escovação dentária e cárie dentária.

**RESULTADOS:**

As médias dos escores do senso de coerência e do apoio social reduziram significativamente entre linha de base e seguimento de dois anos. A incidência de dor dentária no seguimento de dois anos foi 28,6%. O risco de dor dentária foi 14% maior para cada 10 pontos na redução média do escore do senso de coerência (RR = 1,14; IC95% 1,02–1,20), e 6% maior para cada 10 pontos na redução média do escore do apoio social (RR = 1,06; IC95% 1,01–1,11). A mudança na autoestima não foi associada ao risco de dor dentária.

**CONCLUSÃO:**

A variação do senso de coerência e do apoio social no período de dois anos influenciou a incidência de dor dentária em crianças, sugerindo que fatores psicossociais protetores, comportamentos em saúde, plano odontológico e a condição clínica bucal desempenham um papel importante na incidência da dor dentária.

## INTRODUÇÃO

A dor é um evento subjetivo que está relacionado a fatores fisiológicos, psicológicos, sociais e culturais. A dor dentária tem como origem alterações nos dentes ou nas suas estruturas de suporte, sendo a cárie dentária não tratada a sua principal causa biológica^1^ . Diversos fatores como a dieta cariogênica, má higiene bucal, baixa exposição ao flúor e baixo nível socioeconômico têm sido associados à cárie dentária^2^ .

Fatores psicológicos podem exacerbar a percepção da dor devido ao pensamento catastrófico da dor, ansiedade e medo associados à dor, sendo esses os principais fatores psicossociais investigados em relação à dor dentária^3^ . Os fatores psicossociais protetores podem ser definidos como a ausência ou uma baixa exposição a um fator de risco. Apesar de serem conceitualmente distintos dos fatores de risco, eles podem afetar a saúde de forma independente ou atenuar os efeitos dos fatores de risco sobre a saúde^4^ . Fatores psicossociais protetores, como senso de coerência (SOC, do inglês *sense of coherence* ), apoio social e autoestima, podem modular a percepção da dor favorecendo o enfrentamento de eventos de risco^5^ .

A teoria salutogênica tem como base a orientação para resolução dos problemas e a capacidade de identificar e utilizar de forma eficaz os recursos disponíveis para melhorar a saúde, propondo uma compreensão ampliada dos determinantes da saúde ao destacar a importância dos fatores de promoção de saúde e da qualidade de vida da população. O SOC é considerado o constructo central da salutogênese, que compreende ainda os recursos gerais de resistência, incluindo o apoio social e a autoestima^5^ , ele também é definido como uma orientação para que as pessoas percebam a vida como abrangente, gerenciável, significativa^5 , 6^ . Apoio social é o fornecimento de recursos psicológicos e materiais com intenção de ajudar os indivíduos a lidar com o estresse, entenderem que são cuidados, amados e membros de um coletivo^7 , 8^ . Autoestima tem aspectos inter-relacionados que envolvem o senso de eficácia pessoal e de valor pessoal, sendo a soma integrada de autoconfiança e respeito próprio, e a convicção de que o indivíduo é competente para viver, digno de viver^9^ .

Estudos sobre o papel dos fatores psicossociais protetores sobre a saúde bucal, especialmente a dor dentária, são escassos. O objetivo do presente estudo foi investigar a influência de fatores psicossociais protetores, incluindo SOC, apoio social e autoestima, e a incidência da dor dentária nos últimos seis meses em crianças de 12 anos.

## MÉTODOS

Um estudo longitudinal prospectivo foi realizado com escolares, pais e responsáveis na Zona Leste do município de Manaus (AM). Uma amostra aleatória estratificada de crianças de 12 anos, regularmente matriculadas no 7º ano do ensino fundamental, foi selecionada em 25 escolas públicas municipais de 11 bairros da Zona Leste de Manaus. A seleção das escolas foi proporcional ao número de escolas em cada bairro. Crianças com alguma síndrome e/ou que necessitassem de cuidados especiais e que estivessem em tratamento ortodôntico foram excluídas. Esta pesquisa foi aprovada pelo comitê de ética e pesquisa da Universidade Federal do Amazonas (CAAE 57273316.1.0000.5020).

Um modelo teórico com estrutura hierárquica entre os seus componentes foi adaptado para testar a influência dos fatores psicossociais protetores sobre a dor dentária. Nesse modelo, as características sociodemográficas influenciariam o acesso ao plano de saúde odontológico, frequência de consulta odontológica, frequência de escovação dentária e fatores psicossociais protetores, que teriam um efeito sobre as medidas clínicas bucais, incluindo cárie dentária e sangramento gengival, que por sua vez influenciariam a dor dentária ( Figura )^10^ .

**Figura:** Modelo teórico dos preditores para dor dentária adaptado de Bastos et al.10(2005).

A coleta de dados na linha de base foi realizada entre setembro e dezembro de 2016 com um questionário autopreenchível com perguntas fechadas sobre características demográficas, socioeconômicas, plano de saúde odontológico e uso de serviços odontológicos, respondido pelos pais/responsáveis das crianças. Além disso, as crianças responderam a um questionário autoaplicável para avaliar dor dentária e frequência de escovação dentária, além de preencherem as escalas de SOC, apoio social e autoestima validadas para a população brasileira, na linha de base e após dois anos. Examinadores registraram o índice de dentes cariados, perdidos e obturados (CPO-D) e o sangramento gengival durante exame clínico com espelho intrabucal plano n. 5 (Duflex^®^) e sonda OMS tipo *ball point* , e equipamento de proteção individual. O exame clínico bucal foi realizado por cinco (linha de base) e sete (seguimento de dois anos) examinadores previamente calibrados sob luz ambiente e com privacidade em salas de aula escolhidas pela direção de cada escola. Os coeficientes Kappa para calibração interexaminador do índice CPO-D variaram de 0,914 a 0,988.

Avaliou-se a dor dentária nos últimos seis meses com a pergunta: “Nos últimos seis meses você teve dor de dente?” As opções de respostas foram “sim” ou “não”. A intensidade de dor foi avaliada por meio do instrumento *Faces Pain Scale – Revised* (FPS-R)^11^ , na versão adaptada para o português^12^ . O FPS-R é uma escala de faces de dor de autoavaliação, projetada para medir o nível de intensidade da dor percebida a partir de seis faces, apresentadas horizontalmente, que representam diferentes graus de dor, de “nenhuma dor” a “muita dor”.

As características demográficas e socioeconômicas incluíram sexo (masculino/feminino), cor da pele (branca/preta/amarela/parda/indígena), renda familiar mensal em salários mínimos (SM) (≤ ½ SM/> ½ a 1 SM/> 1SM), e escolaridade dos pais (avaliada em anos de estudos concluídos com aprovação). Frequência de escovação dentária (< 3 vezes ao dia/≥ 3 vezes ao dia), plano de saúde odontológico (sim/não) e consulta odontológica nos últimos 12 meses (sim/não) foram avaliados.

O SOC foi avaliado com a escala SOC-13^5 , 13^ . O questionário possui 13 perguntas que são respondidas com uma escala *Likert* de 5 pontos. As pontuações das perguntas que são contrárias ao SOC são invertidas para a obtenção do escore final que é obtido por meio da soma dos pontos alcançados em cada um dos 13 itens. Quanto maior a pontuação, maior o SOC.

O apoio social foi avaliado por meio do instrumento *Social Support Appraisals* (SSA), elaborado especificamente para crianças^14^ . O questionário é composto por 30 itens com seis opções de resposta para cada item: concordo totalmente, concordo bastante, concordo um pouco, discordo um pouco, discordo bastante, discordo totalmente. O escore final do SSA varia de 30 a 180, e quanto maior o escore, maior o apoio social percebido.

A autoestima foi avaliada por meio da Escala de Autoestima que classifica o nível de autoestima em baixo, médio ou alto^15 , 16^ . A escala original foi desenvolvida para adolescentes e possui dez questões fechadas, sendo cinco referentes à “autoimagem” ou ao “autovalor” positivos e cinco se referem à “autoimagem negativa” ou à “autodepreciação”. Os itens são respondidos em uma escala *Likert* de quatro pontos variando entre concordo totalmente, concordo, discordo e discordo totalmente. O maior escore da Escala de Autoestima indica uma maior autoestima.

A cárie dentária foi avaliada conforme o índice CPO-D que mede o ataque de cárie dentária na dentição permanente^17^ . Para isso, os dentes hígidos (código 0), obturados sem cárie (código 3), perdidos (códigos 4 e 5), e com selantes (código 6) foram recodificados como “0”, e os dentes avaliados como cariados (código 1) e restaurados com cárie (código 2) foram recodificados como “1”. Em seguida, os códigos modificados foram somados para obter o número de dentes cariados para cada participante. O número de dentes com sangramento gengival foi calculado a partir da presença ou ausência de sangramento à sondagem. Em cada participante foram examinados um quadrante superior, aleatoriamente selecionado, e o quadrante inferior contralateral correspondente. A presença do sangramento gengival foi registrada em todas as faces de todos os dentes dos quadrantes selecionados a partir do componente sangramento à sondagem do índice CPI, do inglês *Community Periodontal Index* ^18^ .

Inicialmente, a prevalência de dor dentária na linha de base e a incidência de dor dentária e os respectivos intervalos de confiança de 95% (IC95%) foram estimados. A descrição da amostra foi feita a partir de medidas de proporções e médias com IC95% para variáveis independentes, para a amostra na linha de base e no seguimento de dois anos, e segundo a incidência de dor dentária. A mudança na frequência de escovação foi avaliada a partir da diferença entre o número de crianças que relatou escovar os dentes “três vezes ou mais ao dia” entre a linha de base e o seguimento. As diferenças dos escores das escalas SOC-13, SSA e Escala de Autoestima entre a linha de base e o seguimento foram empregadas para estimar a mudança do SOC, apoio social e autoestima, respectivamente.

As correlações entre SOC, apoio social e autoestima na linha de base e no seguimento de dois anos em crianças foi avaliada pelo coeficiente de correlação de Spearman. A frequência de escovação dentária na linha de base e no seguimento de dois anos foi comparada com o teste de McNemar. Comparações dos escores do SOC, apoio social, autoestima e do número de dentes cariados e com sangramento gengival entre a linha de base e o seguimento de dois anos foram avaliadas pelo teste de Wilcoxon para amostras pareadas.

Regressão de Poisson multinível multivariada foi usada para estimar o risco relativo (RR) e IC95% entre as variáveis independentes e incidência de dor dentária. A análise multinível foi empregada em função do processo amostral em dois estágios: escolas e crianças. A associação entre os escores das escalas de SOC, apoio social, autoestima e a incidência de dor dentária foram estimadas para a mudança a cada 10 pontos na escala^19^ . As variáveis independentes com valor de p < 0.10 na análise bruta foram consideradas para a análise multivariada. Em função das correlações significativas entre os fatores psicossociais, modelos de Poisson multinível foram empregados em separado para testar a associação entre cada fator psicossocial e a incidência de dor dentária ajustada para covariáveis.

## RESULTADOS

A amostra da linha de base foi composta por 288 crianças. Durante o seguimento de dois anos do estudo, 78 crianças não foram reavaliadas por perda de seguimento. Assim, a amostra analisada incluiu 210 escolares avaliados após dois anos de seguimento. Dentre eles, 150 não relataram dor dentária nos últimos 6 meses e formaram o grupo controle. O grupo com incidência de dor dentária foi composto por 60 escolares.

A incidência de dor dentária após o seguimento de dois anos foi de 28,6%; a maioria dos escolares com dor dentária assinalou as faces um e dois, que sugerem pouca intensidade da dor dentária percebida (76,7%), a dor intermediária (faces três e quatro) foi escolhida por nove crianças (15%), enquanto a face relativa a “muita dor” foi respondida por cinco crianças (8,3%).

Dentre as crianças com incidência de dor dentária, 60% eram do sexo feminino, 73,3% eram da cor da pele parda, e 45% de famílias com renda mensal entre meio e um salário mínimo. A maioria dos participantes com incidência de dor dentária após os dois anos de seguimento não possuía plano de saúde odontológico (90%), foi ao dentista no último ano (60%) e escovava os dentes menos de 3 vezes ao dia (51,7%). As médias do SOC, apoio social e autoestima nos participantes com incidência de dor dentária foram 47, 145,8 e 31,5, respectivamente. As médias dos escores de SOC, apoio social e autoestima não apresentaram diferenças relevantes entre a amostra total e a amostra estudada na linha de base ( Tabela 1 ).

**Tabela 1:** Características demográficas e socioeconômicas, serviços odontológicos, fatores psicossociais, escovação dentária e medidas clínicas bucais na linha de base, para a amostra total e a amostra estudada, e segundo dor dentária nos últimos seis meses no seguimento de dois anos em crianças.

Variável	Dados da linha de base da amostra estudada (n = 210)	Dor dentária nos últimos 6 meses no seguimento de 2 anos (n = 210)	
		Sim (n = 60)	Não (n = 150)
	Média (IC95%)	Média (IC95%)	Média (IC95%)
Dor dentária no seguimento de 2 anos	28,6 (22,9–35,1)	100,0	0,0
**Características demográficas**			
Sexo			
Masculino	45,2 (38,6–52,1)	40,0 (28,3–52,6)	47,3 (39,4–55,4)
Feminino	54,8 (47,9–61,4)	60,0 (47,0–71,7)	52,7 (44,6–60,6)
Cor da pele			
Branca	16,1 (11,8–21,9)	13,3 (6,7–24,7)	17,3 (12,0–24,3)
Preta	9,1 (5,8–13,8)	5,0 (1,6–14,6)	10,7 (6,6–16,7)
Amarela	3,8 (1,9–7,5)	5,0 (1,6–14,6)	3,3 (1,4–7,8)
Parda	68,1 (61,4–74,1)	73,3 (60,6–83,1)	66,0 (58,0–73,2)
Indígena	2,9 (1,3–6,3)	3,4 (0,8–12,6)	2,7 (0,9–7,0)
**Características socioeconômicas**			
Renda familiar mensal			
≤ ½ SM	25,8 (20,3–32,3)	21,7 (12,9–34,0)	27,5 (20,9–35,3)
> ½ a 1 SM	41,6 (35,1–48,5)	45,0 (32,8–57,8)	40,3 (32,6–48,4)
> 1 SM	32,5 (26,5–39,2)	33,3 (22,5–46,3)	32,2 (25,1–40,2)
Escolaridade dos pais, média	11,1 (9,9–12,3)	11,9 (8,9–14,8)	10,8 (9,5–12,0)
**Serviços odontológicos**			
Plano de saúde odontológico			
Sim	11,4 (7,8–16,5)	21,6 (12,9–34,0)	7,3 (4,1–12,8)
Não	88,6 (81,4–95,2)	90,0 (84,0–93,9)	75,0 (62,4–84,4)
Consulta odontológica nos últimos 12 meses	44,3 (37,7–51,1)	0,60 (0,47–71,7)	47,3 (39,4–55,4)
**Escovação dentária**			
Frequência de escovação dentária			
< 3 vezes ao dia	35,7 (29,5–42,5)	51,7 (39,0–64,1)	29,3 (22,6–37,2)
≥ 3 vezes ao dia	64,3 (57,5–70,5)	48,3 (35,9–61,0)	70,7 (62,8–77,5)
Fatores psicossociais protetores			
Senso de coerência, média	45,8 (45,1–46,5)	47,0 (45,4–48,7)	46,1 (45,2–47,1)
Apoio social, média	144,1 (141,8–146,5)	145,8 (141,3–150,3)	143,5 (140,8–146,2)
Autoestima, média	31,7 (30,9–31,9)	31,5 (30,5–32,4)	31,2 (30,8–31,9)
Medidas clínicas bucais			
Cárie dentária, média	0,5 (0,4–0,7)	0,8 (0,5–1,1)	0,4 (0,2–0,6)
Sangramento gengival, média	3,3 (3,0–3,7)	3,3 (2,5–4,1)	3,4 (2,9–3,8)

IC95%: intervalo de confiança de 95%; SM: salário mínimo.

Todas as correlações entre as variáveis psicossociais na linha de base e entre as variáveis psicossociais no seguimento de dois anos foram estatisticamente significativas. Na linha de base, a correlação mais forte foi entre apoio social e autoestima (coeficiente = 0,419; p < 0,001) e no seguimento de dois anos foi entre SOC e apoio social (coeficiente = 0,632; p < 0,001).

A frequência de escovação dentária ≥ 3 vezes ao dia reduziu de 64,3% para 56,7% entre a linha de base e o seguimento de dois anos. Os escores médios de SOC e apoio social reduziram significativamente entre os períodos. As médias do número de dentes cariados e com sangramento gengival aumentaram de 0,5 para 1,3, e de 3,3 para 8,2, respectivamente, entre a linha de base e o seguimento de dois anos ( Tabela 2 ). Esses resultados indicam uma piora na frequência de escovação dentária, no SOC e no apoio social.

**Tabela 2:** Comparações dos fatores psicossociais, frequência de escovação dentária e medidas clínicas bucais entre a linha de base e dois anos de seguimento em crianças.

Variáveis	Linha de base	Seguimento de 2 anos	p
	Média (IC95%)	Média (IC95%)	
**Escovação dentária**			
Frequência de escovação dentária			0,002^a^
< 3 vezes ao dia	35,7 (29,5–42,5)	43,3 (36,7–50,2)	
≥ 3 vezes ao dia	64,3 (57,5–70,5)	56,7 (49,8–63,3)	
**Fatores psicossociais protetores**			
Senso de coerência, média	45,8 (45,1–46,5)	42,9 (41,7–44,0)	< 0,001^b^
Apoio social, média	144,1 (141,8–146,5)	138,5 (135,6–141,3)	0,001^b^
Autoestima, média	31,7 (30,9–31,9)	30,8 (30,2–31,3)	0,298^b^
**Medidas clínicas bucais**			
Cárie dentária, média	0,5 (0,4–0,7)	1,3 (1,0–1,5)	< 0,001^b^
Sangramento gengival, média	3,3 (3,0–3,7)	8,2 (7,8–8,6)	< 0,001^b^

IC95%: intervalo de confiança de 95%.

^a^p refere-se ao teste de McNemar.

^b^p refere-se ao teste de Wilcoxon para amostras pareadas.

As medidas não ajustadas de risco relativo e os respectivos IC95% entre as variáveis independentes e a incidência de dor dentária nos últimos seis meses no seguimento de dois anos são apresentadas na Tabela 3 . As associações entre incidência de dor dentária após o seguimento de dois anos e plano de saúde odontológico, mudança na frequência de escovação dentária, mudança no SOC, mudança no apoio social e cárie dentária após os dois anos, apresentaram valor de p inferior a 0,10 e foram incluídas nos modelos de regressão de Poisson multinível multivariados. A autoestima não foi associada à incidência de dor dentária no seguimento de dois anos. Portanto, a variável não foi testada no modelo de regressão de Poisson multinível multivariado.

**Tabela 3:** Associações não ajustadas entre características sociodemográficas, serviços odontológicos, escovação dentária, fatores psicossociais protetores, medidas clínicas bucais e incidência de dor dentária em crianças, usando regressão de Poisson multinível.

Variável	RR	IC95%	p
**Características demográficas**			
Sexo (ref.: Masculino)			
Feminino	1,24	0,74–2,10	0,416
Cor da pele (ref.: Branca)			
Preta	0,70	0,18–2,69	0,606
Amarela	1,69	0,44–6,54	0,449
Parda	1,34	0,63–2,88	0,449
Indígena	1,49	0,31–7,18	0,621
**Características socioeconômicas**			
Renda familiar mensal (ref.: > 1 SM)			
> ½ a 1 SM	1,08	0,60–1,94	0,797
≤ ½ SM	0,83	0,41–1,69	0,609
Escolaridade dos pais	1,01	0,99–1,03	0,512
**Serviços odontológicos**			
Plano de saúde odontológico (ref.: Não)			
Sim	0,46	0,25–0,87	0,017^b^
Consulta odontológica nos últimos 12 meses (ref.: Não)			
Sim	0,96	0,57–1,60	0,864
**Escovação dentária**			
Frequência de escovação dentária (2 anos) (ref.: < 3 vezes ao dia)			
≥ 3 vezes ao dia	0,88	0,53–1,47	0,616
Mudança na frequência de escovação dentária	1,52	0,95–2,44	0,078^b^
Fatores psicossociais protetores			
Senso de coerência (linha de base)^a^	1,15	0,86–1,19	0,449
Senso de coerência (2 anos)^a^	0,87	0,69–1,03	0,111
Mudança no senso de coerência^a^	1,13	1,01–1,19	0,035^b^
Apoio social (linha de base)^a^	1,03	0,95–1,09	0,488
Apoio social (2 anos)^a^	0,96	0,89–1,02	0,153
Mudança no apoio social^a^	1,05	1,00–1,10	0,053^b^
Autoestima (linha de base)^a^	1,04	0,63–1,21	0,825
Autoestima (2 anos)^a^	0,84	0,51–1,12	0,312
Mudança na autoestima^a^	1,11	0,87–1,20	0,319
**Medidas clínicas bucais**			
Cárie dentária (linha de base)	1,14	0,97–1,34	0,109
Cárie dentária (2 anos)	1,11	0,99–1,24	0,075^b^
Mudança na cárie dentária	1,08	0,91–1,29	0,369
Sangramento gengival (linha de base)	0,99	0,90–1,09	0,824
Sangramento gengival (2 anos)	0,96	0,89–1,05	0,377
Mudança no sangramento gengival	1,02	0,95–1,09	0,579

RR: risco relativo; IC95%: intervalo de confiança de 95%; SM: salário mínimo.

^a^Estimativas para cada 10 pontos na escala.

^b^p < 0,10.

Modelos de regressão de Poisson multiníveis multivariados entre fatores psicossociais protetores e a incidência de dor dentária foram analisados em separado para SOC e apoio social devido à colinearidade observada entre as variáveis ( Tabela 4 ). A mudança a cada 10 pontos na escala de SOC aumentou em 14% o risco de dor dentária (RR = 1,14; IC95% 1,02–1,20). A incidência de dor dentária foi 6% maior a cada aumento de 10 pontos na mudança do escore da escala de apoio social (RR = 1,06; IC95% 1,01–1,11). Escolares com plano de saúde odontológico tiveram menor incidência de dor dentária em relação aos sem plano de saúde odontológico. Além disso, o risco para dor dentária nos últimos dois anos foi maior entre crianças que modificaram a frequência de escovação em comparação àquelas que não modificaram.

**Tabela 4:** Modelos de regressão de Poisson multinível entre plano de saúde odontológico, mudança na frequência de escovação dentária, SOC e apoio social, cárie dentária e incidência de dor dentária.

Variável	RR	IC95%	p
**Senso de coerência (Modelo ajustado)**			
Plano de saúde odontológico (ref.: Não)			
Sim	0,49	0,26–0,91	0,024
Mudança na frequência de escovação dentária	1,61	1,01–2,57	0,046
Senso de coerência na linha de base^a^	0,96	0,68–1,15	0,745
Mudança no senso de coerência^a^	1,14	1,02–1,20	0,030
Cárie dentária no seguimento de 2 anos	1,10	0,99–1,22	0,087
**Apoio social (modelo ajustado)**			
Plano de saúde odontológico (ref.: Não)			
Sim	0,47	0,25–0,87	0,017
Mudança na frequência de escovação dentária	1,69	1,04–2,76	0,035
Apoio social na linha de base^a^	0,99	0,90–1,06	0,780
Mudança no apoio social^a^	1,06	1,01–1,11	0,038
Cárie dentária no seguimento de 2 anos	1,08	0,97–1,20	0,172

SOC: do inglês *sense of coherence* ; RR: risco relativo; IC95%: intervalo de confiança de *95%* .

^a^Estimativas para cada 10 pontos na escala.

As estimativas das variáveis independentes estão ajustadas entre si.

## DISCUSSÃO

Esse estudo avaliou o possível efeito de mudanças em fatores psicossociais protetores, incluindo SOC, apoio social e autoestima, sobre a incidência da dor dentária em crianças no período de dois anos. Reduções significativas foram observadas no SOC e apoio social entre a linha de base e após dois anos de seguimento. A redução do SOC pode estar relacionada à condição social dos participantes, pois um estudo anterior relatou que o SOC reduziu em adultos com piores indicadores sociais^20^ . A autoestima não variou entre os períodos de estudo. As variações no SOC e apoio social aumentaram o risco para a incidência da dor dentária em crianças.

A importância do acesso a plano de saúde odontológico e comportamentos, incluindo frequência de escovação dentária, para a ocorrência da dor dentária foi confirmada neste estudo^2^ . No entanto, a associação entre cárie dentária e dor dentária não foi observada no presente estudo, apesar de ter sido relata em estudos anteriores^1 , 2^ . Uma possível explicação para o resultado foi o emprego do índice CPO-D para avaliar a cárie. O índice CPO-D não mensura a gravidade da cárie, apenas a sua presença ou ausência. Assim, este resultado pode ter ocorrido porque uma significativa proporção de dentes cariados na amostra era composta por lesões iniciais de cárie dentária.

Estudos sobre a possível influência dos fatores psicossociais na dor dentária em crianças são escassos. Uma revisão sistemática prévia demonstrou que medo e a ansiedade relacionados ao atendimento odontológico são comuns em crianças e adolescentes e que esses fatores psicológicos foram relacionados com a dor dentária^3^ . Ademais, o estresse e a ansiedade foram preditores para a expectativa, percepção e memória da dor dentária em crianças^21^ . A ansiedade foi associada ao início da dor orofacial durante um acompanhamento de dois anos^22^ . São escassas as evidências da influência dos fatores psicossociais sobre a dor dentária, considerando a teoria salutogênica^23^ .

Existem evidências da relação entre fatores psicossociais protetores e desfechos em saúde bucal e uso de serviços odontológicos. O maior SOC materno aumentou a probabilidade do uso de serviços odontológicos dos seus filhos por motivos preventivos e de revisão^24^ . Revisão sistemática recente evidenciou a relação entre fatores psicossociais protetores, incluindo o SOC materno, autoeficácia e apoio social, e a menor probabilidade de cárie dentária em crianças e adolescentes^4^ . A associação entre SOC materno e menor ocorrência de cárie em crianças também foi demonstrada^25^ . Além disso, o SOC, autoestima e crenças em saúde em adolescentes foram preditores diretos para a cárie dentária não tratada e para a qualidade de vida relacionada à saúde bucal^26^ . Em outro estudo, o maior apoio social foi associado à melhor condição clínica bucal em adolescentes^27^ . Além disso, o apoio social teve um efeito indireto sobre a dor dentária e essa relação foi mediada pela condição clínica bucal. O apoio social influenciou ainda a qualidade de vida relacionada à saúde bucal e a percepção da saúde bucal^27^ . Assim, a associação encontrada em fatores psicossociais e dor dentária pode ser explicada pelos efeitos positivos dos fatores psicossociais protetores sobre o uso de serviços odontológicos e condições clínicas bucais.

Apesar da autoestima não ter sido associada à dor dentária neste estudo, a autoestima atenuou o impacto da má oclusão na qualidade de vida relacionada à saúde bucal em crianças com menor necessidade de tratamento ortodôntico. Além disso, a autoestima foi associada à qualidade de vida relacionada à saúde bucal^9^ . Pode-se se sugerir que a autoestima seja um fator psicossocial relevante para medidas subjetivas bucais, mas que não seja suficiente para influenciar a dor dentária.

A possível relação entre fatores psicossociais e dor dentária tem sido avaliada em estudos epidemiológicos seccionais. Pesquisas longitudinais envolvendo fatores psicossociais protetores e desfechos em saúde bucal foram encontradas sobre a relação entre SOC, apoio social e crenças com saúde bucal, a qualidade de vida relacionada à saúde bucal e comportamentos em saúde bucal^26 , 28^ . Nenhum estudo longitudinal foi encontrado sobre fatores psicossociais protetores e dor dentária. Assim, destaca-se a necessidade de mais estudos longitudinais nesse tema para aumentar a compreensão dos determinantes da dor dentária, incluindo o papel mediador e moderador dos fatores psicossociais, da relação entre características socioeconômicas e clínicas com a dor dentária. Estudo prévio revelou que o SOC, apesar de não ter sido diretamente relacionado à dor dentária, teve um papel moderador relevante na associação entre cárie dentária e dor dentária^23^ .

A busca na resolução de problemas, o uso de recursos que direcionam o indivíduo a comportamentos mais saudáveis em saúde, um senso coerente que faça perceber a vida como gerenciável e significativa são formas de modulação desempenhadas pelo SOC segundo a perspectiva da abordagem salutogênica. Assim, um forte SOC permite que as pessoas mantenham os cuidados em saúde bucal, mesmo diante de situações de vida estressantes^6^ . O apoio social, mediante o estabelecimento das conexões sociais, fornece recursos emocionais, materiais e psicológicos para enfrentamento de situações adversas e estressantes^8^ . As relações mútuas entre indivíduos podem ainda influenciar positivamente comportamentos em saúde por controle e pressão social, além de gerar um sentimento de responsabilidade, o que motiva e fortalece o autocuidado e o cuidado mútuo entre membros de grupos sociais. Por exemplo, participar de uma rede social na qual seus membros possuem hábitos de higiene bucal e alimentação saudável, influencia o sujeito a seguir os mesmos comportamentos saudáveis do grupo, tendo, assim, impactos positivos sobre a saúde^7^ . Esses elementos reforçam a importância da abordagem salutogênica e seus princípios na fundamentação para formulação de políticas e estratégias de promoção da saúde.

A maioria das ações coletivas em saúde bucal propõe apenas medidas preventivas individuais e coletivas, como o aumento no acesso ao uso do flúor por meio do creme dental fluoretado e da fluoretação da água de abastecimento para prevenção da cárie dentária. Além disso, prevalecem as intervenções individuais centradas em mudanças comportamentais, incluindo aumento na frequência da escovação dentária e melhoria nas técnicas de escovação. Dessa forma, a implementação de estratégias voltadas para a promoção de saúde bucal, aliadas à perspectiva salutogênica, podem ser consideradas incipientes. Recente revisão sistemática demonstrou que intervenções sobre as redes sociais modificou positivamente comportamentos, incluindo a redução do tabagismo, e a percepção de bem-estar^29^ . Estudo de intervenção concluiu que o aumento do SOC resultou na melhoria da qualidade de vida relacionada à saúde bucal em crianças^30^ . Assim, futuros estudos de intervenção deverão abordar a possibilidade da redução da percepção da dor dentária a partir da modificação de fatores psicossociais protetores.

Contudo, algumas limitações deste estudo devem ser consideradas. A amostra do estudo incluiu crianças com 12 anos na linha de base, que foram acompanhadas por dois anos, portanto, os achados não devem ser considerados para outros grupos etários. Os participantes foram selecionados em uma área de desigualdade social. Assim, sugere-se cautela na extrapolação dos resultados para populações que vivem em condições sociais diferentes. Por fim, alguns fatores comportamentais relacionados à dor dentária propostos no modelo teórico adotado neste estudo, incluindo uso de creme dental e uso de fio dental, não foram avaliados^9^ .

Dentre os fatores psicossociais protetores investigados no presente estudo, os escores do SOC e apoio social reduziram entre a linha de base e os dois anos de seguimento. Observou-se ainda uma redução na frequência da escovação dentária. A variação do SOC e do apoio social aumentaram significativamente o risco para dor dentária no seguimento de dois anos. A redução na frequência da escovação dentária e a ausência de plano de saúde odontológico também influenciaram a incidência da dor dentária. Os achados deste estudo sugerem que fatores psicossociais protetores, comportamentos em saúde e plano de saúde odontológico desempenham papel importante na incidência da dor dentária em crianças.
